# Renal Calculi

**Published:** 1889-12-21

**Authors:** 


### RENAL CALCULI.

"On the Removal of Renal Calculi by Toxic Doses of Belladonna" (*Provincial Medical Journal*) is the title of a paper by Dr. W. Murray, of Newcastle. Three cases to the point are recorded. Tincture of belladonna in from twenty to forty drops every hour, until toxic effects such as dilatation of the pupil, dryness of the throat, and delirium were produced. In the first case, when the patient was fully under the influence of the drug, a lithic acid calculus as large as an almond passed "with the usual satisfactory click." In the second case, the treatment was commenced in the afternoon, and during the night a calculus as large as a bean was passed. In the third case, operation had been proposed, but at the end of four or five hours' treatment a round and rough calculus was passed. These cases, Dr. Murray maintains, are sufficiently striking to arrest attention. The special point to be remembered is, that the drug is not only to be pushed to its toxical stage, but its action is to be kept up after pain has been relieved, until a fair time has been allowed for the expulsion of the stone. Dr. Murray appears to have reasoned from analogy with regard to the action of belladonna on the urinary passages. He was led to infer that something more than relief of pain might be expected by considering the analogous condition of bowel obstruction in which belladonna has proved so successful. It has not, however, yet been satisfactorily ascertained in what manner belladonna affects the intestines. Some have asserted that belladonna increases the peristaltic movement of the canal; others have asserted that the drug weakens this action. Probably the correct view is that it in small doses increases peristalysis, but in large doses it paralyses the muscular tissue of the intestine. Ringer tells us that it has been experimentally proved that belladonna paralyses the terminations of the inhibitory fibres of the splanchnics distributed to the intestines. The whole subject of the action of belladonna appears to be involved in more or less obscurity. If we recollect right, Dr. Weir Mitchell, Philadelphia, and Dr. Da Costa, Pennsylvania, have obtained marked results in spasmodic nervous affections from the employment of belladonna, and they regard the benefit as due to the depressing effect of the drug. Wood in "Therapeutics, and Principles of Practice," says that whenever possible it should be used locally. Thus in spasm of the urethra, belladonna ointment may be rubbed in along the canal; in rigid *os uteri* the extract should be applied to the *os*; in asthma, belladonna should be inhaled; in spasm of the *sphincter ani* it should be applied to the part. Should further experience confirm Dr. Murray's plan of using belladonna, a great advance will be made in the treatment of a very painful and dangerous disorder.

